# Severe vaso-occlusive lupus retinopathy in the early stage of a pediatric patient with systemic lupus erythematosus: a case report

**DOI:** 10.1097/MD.0000000000019875

**Published:** 2020-04-17

**Authors:** Guoping Huang, Huijun Shen, Jingli Zhao, Jianhua Mao

**Affiliations:** Department of Nephrology, The Children's Hospital Zhejiang University School of Medicine, Hangzhou, Zhejiang Province, China.

**Keywords:** autoimmune disease, lupus retinopathy, systemic lupus erythematosus (SLE), vaso-occlusive retinopathy

## Abstract

**Introduction:**

Systemic lupus erythematosus (SLE) is a multisystem, chronic, autoimmune disease which can affect any organ system including the eye. About one-third of the patients can be diagnosed with SLE-related eye involvement which is usually indicative of disease activity. Retinopathy is one of the most vision-threatening complications that can be associated with the disease.

**Patient concerns:**

An 11-year-old girl was hospitalized with complains of repeated swelling and pain in her extremities for 1 month, chest pain for 24 days, rash for 5 days and proteinuria for 1 day. On the morning of her fourth day in hospital, she suddenly complained of sudden, painless vision loss in the left eye. The ophthalmologist found that she had obstruction of central retinal vein and artery with diffuse retinal hemorrhages and macular edema.

**Diagnosis:**

The patient was diagnosed with systemic lupus erythematosus, lupus nephritis, and lupus retinopathy through her clinical manifestations and laboratory tests.

**Interventions:**

After diagnosis, she received steroid therapy, retinal laser photocoagulation, and intravitreal injection of dexamethasone (OZURDEX, Allergan Pharmaceuticals, Dublin, Ireland) early in her course.

**Outcomes:**

At the latest follow-up, her vision improved partially. However, she still has the possibility of subsequent neovascular glaucoma and bleeding in the future.

**Conclusions:**

An early diagnosis and the prompt therapeutic measures are necessary to prevent sight-threatening consequences, especially in pediatric patients with SLE.

## 1 Introduction

Systemic lupus erythematosus (SLE) is a multisystem, chronic, autoimmune disease which can affect any organ system including the eye. The incidence is much higher in women than in men (female/male ratio ranging from 6:1 to 10:1). African women have the highest incidence and prevalence in their childbearing age.^[1,2]^ SLE affects roughly 20 to 150 people per 100,000.^[3]^ While Pediatric-onset SLE (pSLE) is rare with a prevalence of 3.3 to 8.8 per 100,000 children.^[4]^ The etiology of SLE is still undefined, but variably intertwined factors such as genetic predisposition, environmental stimuli, and an unfailing dysregulation of the immune system play a role together.^[5,6]^

Ocular manifestations which could occur at any stage of the disease can be found in approximately one-third of SLE patients, and can affect any part of the visual system, and are usually indicative of disease activity.^[7]^ Keratoconjunctivitis sicca or secondary Sjögren syndrome is the most frequent ophthalmic manifestation of SLE. While retinal involvement is the second frequent one.^[7]^ The incidence of SLE-associated retinopathy has been reported to be 7% to 29% among adult patients. Occlusive retinopathy has been found in approximately 3% to 11%. ^[8,9]^ Although no relevant studies have been reported to our knowledge, retinopathy appears to be less common among children with SLE. Due to its infrequency we report a case of severe vaso-occlusive lupus retinopathy in the early stage of a pediatric patient with SLE.

## 2 Case report

An 11-year-old Chinese girl was admitted to a childrens hospital in December, 2018. Her complains were swelling and pain in her extremities for 1 month, chest pain for 24 days, rash for 5 days and proteinuria for 1 day. She had no family history of genetic or similar diseases. After her admission, She was found to be antinuclear antibodies (ANA) positive (1:320 on immunofluorescence assay), with positive antibodies to double-stranded DNA(dsDNA), nucleosome, and cardiolipin. Her chest CT showed pleural effusion and a little pericardial effusion. Her complement levels of C3 and C4 were decreased, accompanied by proteinuria of 376 mg/day. The day after her hospitalization, she was checked by the ophthalmologist and at that time no abnormal ocular manifestations were found. Combined with her clinical manifestations with the exclusion of tumors, drugs or infections, this patient was clearly diagnosed with SLE and lupus nephritis on the third day after hospitalization according to the 2012 Systemic Lupus International Collaborating Clinics (SLICC) classification criteria.^[10]^

**Figure 1:**
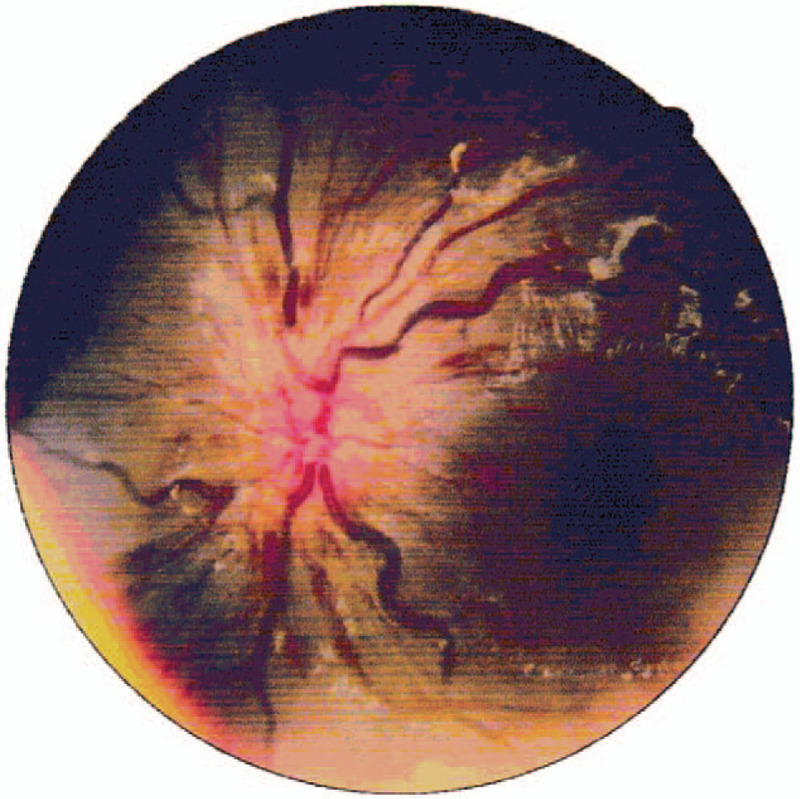
Fundus photograph (21/12/2018). left eye: obstruction of central retinal artery and vein with diffuse retinal hemorrhages, tortuous dilatation of vessels and edema of optic disc.

**Figure 2:**
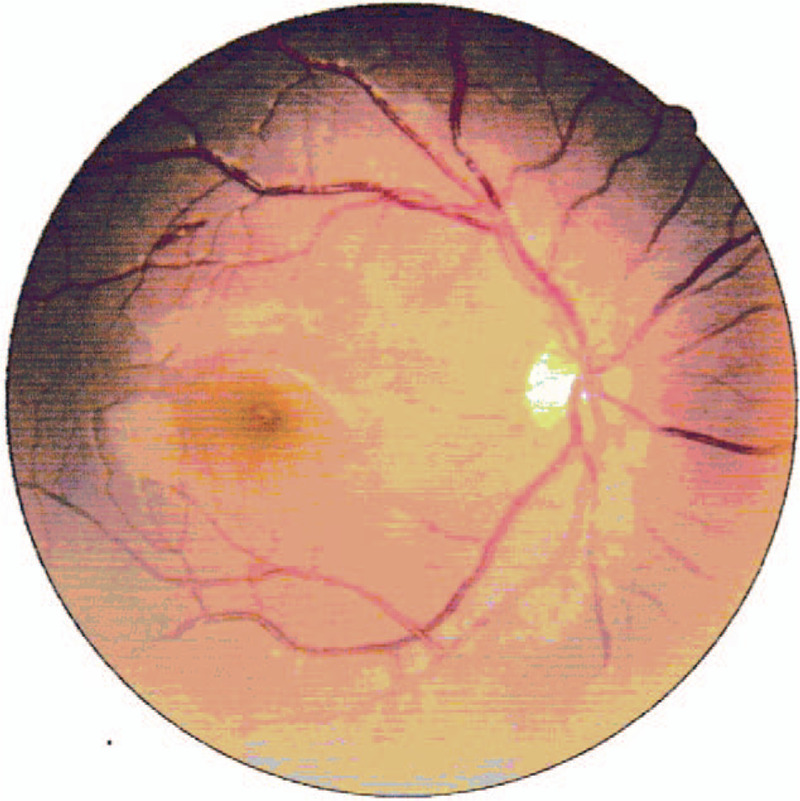
Fundus photograph (21/12/2018). right eye: no changes.

**Figure 3:**
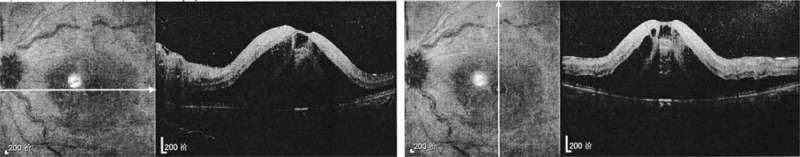
Spectral domain optical coherence tomography (SD-OCT, Heidelberg Engineering, Heidelberg, Germany) of the left eye (21/12/2018) showed severe macular edema and uplifted. It also showed massive effusion between the outer layers of the neuroretina.

Oral hydroxychloroquine sulfate and low dose of intravenous methylprednisolone therapy were prescribed immediately after diagnosis. On the morning of her fourth day in hospital, the patient complained of sudden, painless vision loss in the left eye. Because the ophthalmology department of the childrens hospital lacked some equipment, the patient immediately went to another general hospital for ophthalmological examination. On examination her visual acuity was 20/20 in the right eye and hand motion at 10 cm in the left eye. The intraocular pressure was normal, 17 mm Hg in the right eye and 14 mm Hg in the left eye. Funduscopic examination (FE) showed obstruction of central retinal artery and vein with diffuse retinal hemorrhages, tortuous dilatation of vessels, and edema of optic disc in the left eye while no changes in the right eye (Figs. 1 and 2). Spectral domain optical coherence tomography (SD-OCT, Heidelberg Engineering, Heidelberg, Germany) indicated severe macular edema and uplifted (Fig. 3). It also showed massive effusion between the outer layers of the neuroretina. The diagnosis was lupus retinopathy. The pediatrician conducted the first course of intravenous steroid pulse therapy (methylprednisolone 15 mg/kg/day × 3 days) followed by low dose of intravenous methylprednisolone on the advice of the ophthalmologist in order to decrease disease activity and prevent visual loss.

On the eighth day after her hospitalization, the patient returned to the ophthalmologist. Unfortunately, there was no improvement in her left eye. The intraocular pressure was 17 mm Hg in the right eye and 12 mm Hg in the left eye. And the visual acuity was still hand motion in the left eye. FE and SD-OCT showed the same findings as those on the fourth day. So, the ophthalmologist suggested that the patient next undergo retinal laser photocoagulation and intravitreal dexamethasone (OZURDEX, Allergan Pharmaceuticals, Dublin, Ireland) in the left eye. During the following week, she underwent the second course of intravenous steroid pulse therapy, laser photocoagulation and intravitreal dexamethasone(OZURDEX, Allergan Pharmaceuticals, Dublin, Ireland) in her left eye. Her urine protein turned negative immediately.

**Figure 4:**
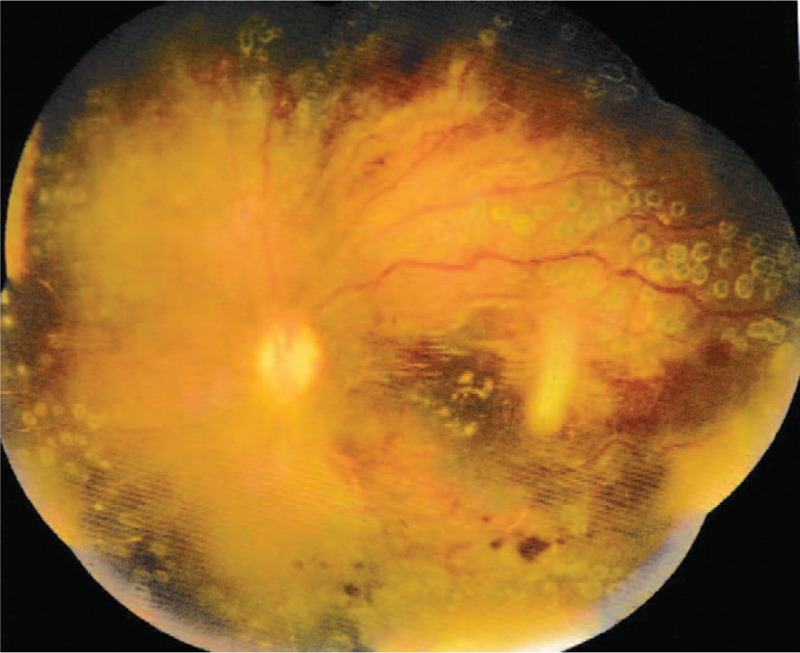
Fundus photograph of the left eye (29/4/2019) showed improved vascular tortuosity, resorption of retinal hemorrhages, and reduced optic disc edema.

**Figure 5:**
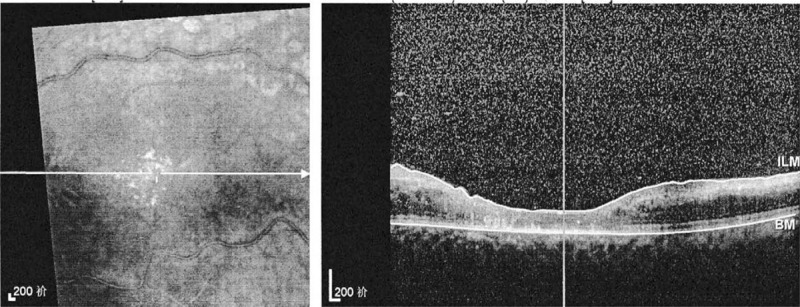
Spectral domain optical coherence tomography (SD-OCT, Heidelberg Engineering, Heidelberg, Germany) of the left eye (29/4/2019) improvement in macular edema and uplifted.

After 3 weeks, her anti-cardiolipin (ACL) antibody turned negative. During the next 4 months, this patient took low dose methylprednisolone and mycophenolate mofetil. This patient regularly followed up at the ophthalmology and pediatric nephrology clinic. At the latest follow-up, her visual acuity was 20/125 in the left eye. FE showed improved vascular tortuosity, resorption of retinal hemorrhages, and reduced optic disc edema in the left eye (Fig. 4). And some of the blocked vessels showed perfusion. SD-OCT indicated improvement in macular edema and uplifted (Fig. 5). During the 4 months, her intraocular pressure had risen to 29 mm Hg. Under the effect of topical medications (beta- adrenoceptor blocker, alpha2- adrenoceptor agonist and carbonic anhydrase inhibitor), her intraocular pressure returned to normal. However, she still has the possibility of subsequent neovascular glaucoma and bleeding in the future.

## 3 Discussion

The first report of lupus in the eye was in 1929. And in 1933, Semon and Wolff, described the histopathological characteristics of choroiditis and subretinal exudation.^[11]^ Ocular involvement may correlate with systemic disease activity and precede other systemic symptoms.^[12]^ Unilateral or more often bilateral retinopathy is responsible for visual loss of variable severity.^[7]^

The major pathology of lupus retinopathy is thought to be an immune complex-mediated vasculopathy. It is attributed to microangiopathy commonly.^[13,14]^ There are 2 ways that autoimmune processes can affect the retina: directly, by immune complex-mediated vasculitis, and indirectly, by secondary hypertension from renal involvement. And there are 3 types of direct retinal damage by lupus: microangiopathy, severe vaso-occlusion and vasculitis.^[15]^ Among the 3 types, microangiopathy is the mild form. The classic retinal findings include cotton wool spots, microaneurysms, hard exudates and dot hemorrhages.^[16,17]^ Small intraretinal hemorrhages and cotton-wool spots account for 80% of cases and are usually associated with a good visual prognosis.^[8]^

The classic sign of vasculitis is vascular sheathing, which can present in arterioles and/or venules. Vaso-occlusion is a common end-point of vasculitis that may alter visual function.^[15]^

Severe vaso-occlusion is the most severe form of lupus retinopathy. Severe vaso-occlusive retinopathy is associated with widespread retinal capillary nonperfusion, multiple branch retinal artery occlusions, ocular neovascularization, vitreous hemorrhages, tractional retinal detachment, neovascular glaucoma, and significant resultant visual loss.^[18,19]^ Central retinal vein or artery occlusions can also occur, either independently or together, and may be unilateral or bilateral.^[18]^ A study by Jabs et al disclosed 55% of eyes with severe retinal vaso-occlusive disease suffered vision loss, often due to a visual acuity of worse than 20/200.^[20]^ Visual acuity recovery was usually poor despite prompt treatment.^[21]^

In addition, some studies have demonstrated that the correlation between anti-phospholipid antibody syndrome (APS) and retinopathy. In these patients the antibodies that characterize APS are frequently found to be positive, such as lupus anticoagulant (LAC), ACL, and anti-beta-2 glycoprotein-I antibodies, usually of IgG isotype. And it is well known that recurrent infarctions and thromboembolisms are the hallmark of the APS.^[22]^ In this patient, her anti-cardiolipin was positive, this might be related with her severe vaso-occlusive retinopathy.

Visual prognosis of retinal involvement depends on pattern of retinopathy, and vaso-occlusion usually leads to poor visual outcome.^[15]^

As retinopathy is often associated with the activity of SLE, treating the systemic disease may result in improvement of retinopathy. The systemic therapy is based on the typical combination (glucocorticoids[GCs], hydroxychloroquine, and immunosuppressive drugs). The effective immunosuppressive drugs include azathioprine, methotrexate, mycophenolate mofetil, and cyclophosphamide. During the past 20 year, the treatment of SLE is remarkably improved. Increasingly, patients with lupus who do not respond to conventional immunosuppressive drugs are considered for targeted biological therapies aimed at cytokines, B and T lymphocytes, and B-cell-activating factors. Rituximab, B-cell-depleting therapy, has been used when conventional drugs have proven ineffective.^[23]^ Belimumab, a monoclonal human antibody that inactivates B-cell-activating factor, is the first biologic recently approved by the FDA after 50 years as an add-on therapy for active SLE.^[23,24]^ The Clinical activity profile supports the continued clinical development of Sifalimumab, a human anti-interferon-α monoclonal antibody, in the treatment of patients with SLE.^[23]^ Epratuzumab, a CD22-targeted monoclonal antibody, was reportedly associated with improvements in the treatment of adult SLE.^[25]^ If anti-phospholipid antibodies are detected, the addition of a single or dual anti-platelet therapy, or anti-coagulation with warfarin, or any of the novel oral anticoagulants may be useful.^[7]^ Adverse events(AE) of the above mentioned drugs which can affect several organs, including ocular toxicity are also widely observed. GCs long-term administration inevitably results in a well-known series of AE such as diabetes, osteoporosis, and fractures and includes ocular manifestations such as posterior subcapsular cataracts and secondary open-angle glaucoma.^[26]^ The long-term administration of hydroxychloroquine may be associated with AE such as vortex keratopathy and in particularly the dreadful maculopathy. No remarkable adverse effects involving the eye have been reported so far for azathioprine, mycophenolate mofetil, and tacrolimus. The same is true with rituximab and bevacizumab.^[7]^

In addition to systemic therapy, local targeted therapies for SLE retinopathy may also be of benefit. Laser photocoagulation has been known as standard treatment in ischemic retinal disorders such as diabetic retinopathy and ischemic retinal vascular occlusion. Panretinal photocoagulation showed a good effect in regression of neovascularization before the anti-vascular endothelial growth factor (anti-VEGF) era.^[18]^ Intravitreal anti-vascular endothelial growth factor injections can be highly effective in reducing macular edema and retinal neovascularization. Dexamethasone implants have shown promising results for the treatment of macular edema.^[27]^ Besides, vitrectomy is also performed to halt neovascularization and prevent aggravation of visual loss.

In this case, we describe an 11-year-old pediatric patient with lupus retinopathy in the early stage of SLE. As it is said above, pediatric-onset SLE (pSLE) is rare compared with adult-onset SLE. Although no relevant studies have been reported to our knowledge, retinopathy appears to be less common among children with SLE. Up to now, studies on SLE-associated retinopathy, especially on occlusive retinopathy, have focused on adult patients. Due to its infrequency many pediatricians have a poor understanding of SLE-associated retinopathy in children. Therefore, case analysis of it is very important for us to accumulate clinical experience. On the morning of the patients fourth day in hospital, she complained of sudden, painless vision loss in the left eye. The ophthalmological examination showed obstruction of central retinal artery and vein with diffuse retinal hemorrhages, tortuous dilatation of vessels and edema of optic disc in the left eye. So the girl had the most severe form of lupus retinopathy—severe retinal vaso-occlusion. A study by Jabs et al disclosed 55% of eyes with severe retinal vaso-occlusive disease suffered vision loss, often due to a visual acuity of worse than 20/200. ^[20]^ Visual acuity recovery was usually poor despite prompt treatment.^[21]^ Fortunately, unlike the patients with poor prognosis mentioned in the literature above, she received prompt and effective combination therapy which included systemic treatment and local treatment. After 4 months of follow-up, her vision in the left eye was partially restored. Her visual acuity was 20/125. Visual prognosis of retinal involvement depends on different patterns of retinopathy. But this case reminds us the early recognition of retinal changes as well as early ophthalmologic intervention are also important in better outcomes. On the other hand, the childrens vascular wall has good elasticity and abundant microcirculation, which may also be influencing factors for the good prognosis of the patient. However, the patient still needs regular follow-up in the future. If neovascularization was found and the visual acuity deteriorated during the follow-up, panretinal photocoagulation and vitrectomy might be needed.

In fact, we do not pay enough attention to ophthalmic involvement in patients with SLE, especially in pediatric patients. No matter in the American College of Rheumatology (ACR) classification criteria for SLE in 1982,^[28]^ revised in 1997^[29]^ or the Systemic Lupus International Collaborating Clinics (SLICC) classification criteria in 2012,^[10]^ there is no description of ophthalmic involvement. In fact, ophthalmic involvement can represent the initial features of SLE; meanwhile, a delayed diagnosis and the consequent late beginning of treatment may result in sight-threatening consequences. A professional medical team for SLE patients needs to include ophthalmologists. So, we strongly recommend that all SLE patients should undergo a thorough ophthalmic examination at diagnosis. Even if these patients do not show ocular manifestations initially, they might have ocular adverse events after chronic therapy with GCs and/or hydroxychloroquine. So, they should also adhere to eye monitoring.

## Author contributions

**Conceptualization:** Guoping Huang.

**Data curation:** Guoping Huang.

**Formal analysis:** Guoping Huang.

**Investigation:** Guoping Huang.

**Methodology:** Huijun Shen, Jingli Zhao.

**Resources:** Huijun Shen.

**Supervision:** Jianhua Mao.

**Writing – original draft:** Guoping Huang.
